# HIV-2 infection and HIV-1/HIV-2 dual reactivity in patients with and without AIDS-related symptoms in Gabon.

**DOI:** 10.3201/eid0401.980123

**Published:** 1998

**Authors:** C Tevi-Benissan, M Okome, M Makuwa, M N Nkoume, J Lansoud-Soukate, A Georges, M C Georges-Courbot, L Belec


2. Glezen WP. Emerging infections: pandemic influenza. Epidemiol Rev 1996;18:64-76.
3. Patriarca PA, Cox NJ. Influenza pandemic preparedness plan for the United States. J Infect Dis 1997;176(Suppl 1):S4-S7.
4. Hampson AW. Surveillance for pandemic influenza. J Infect Dis 1997;176(Suppl 1):S8-S13.
5. Gambel JM, Hibbs RG. U.S. military overseas medical research laboratories. Mil Med 1996;161:638-45.
6. Williams RJ, Cox NJ, Regnery HL, Noah DL, Khan AS, Miller JM, ET AL. Meeting the challenge of emerging pathogens: the role of the United States Air Force in global influenza surveillance. Mil Med 1997;162:82-6.
7. Rota PA, Hemphill ML, Whistler T, Regnery HL, Kendal AP. Antigenic and genetic characterization of the hemagglutinins of recent circulating strains on influenza type B virus. J Gen Virol 1992:73:2737-42.
8. Centers for Disease Control and Prevention. Update influenza activity—United States and worldwide, 1996-97 season, and composition of the 1997-98 influenza vaccine. MMWR Morb Mortal Wkly Rep 1997;46:325-30.

### HIV-2 Infection and HIV-1/HIV-2 Dual Reactivity in Patients With and Without AIDS-Related Symptoms in Gabon

**To the Editor**: Between 1996 and 1997, we evaluated the incidence of HIV-2 infection at the Fondation Jeanne Ebori, the second largest hospital in Libreville, capital of Gabon; we found an unexpected high prevalence of HIV-2–infected or HIV-1/HIV-2–dually reactive patients.

During a 10-month period, 147 (14.3%) of 1,029 sera from inpatients and outpatients were found HIV-positive by the type III method recommended by the World Health Organization (two enzyme-linked immunosorbent assays are used to screen anti-HIV antibodies) (1). Further discrimination between HIV-1 and HIV-2 infections was assessed by using synthetic peptides specific for the gp41 and the gp120 of HIV-1 and the gp36 of HIV-2 (ImmunoComb II, PBS Orgenics, Illkirch, France). Of the 147 HIV-positive sera, 141 (96.0%) were exclusively HIV-1–positive; four were exclusively HIV-2–positive; and two were both HIV-1– and HIV-2–positive. Of the six sera with anti-gp36/HIV-2 reactivities, two (from patients A and B) were positive on HIV-2 Western blot, with marked anti-gag HIV-1 cross-reactivity and a discrimination assay positive only for HIV-2; two (from patients D and E) were positive on HIV-2 Western blot, with anti-gag and pol reactivities markedly lower than anti-env reactivities and a discrimination assay positive only for HIV-2; the two remaining sera (from patients C and F) showed typical dual reactivities for HIV-1 and HIV-2 infections, with positive patterns of HIV-1 and HIV-2 Western blots and a discrimination test positive for both viruses. As a whole, six (4.1%) of 147 HIV-positive sera showed either HIV-2 infection alone (n = 4) or dual reactivity. Of those, four were from Gabonese patients B, C, D, and E, and two were from immigrants from West Africa (patient A from Mali and patient F from Nigeria); two were female patients B and E. Among Gabonese patients, only one (patient E) had traveled to West Africa; the remaining three had never visited any neighboring country. However, one Gabonese man (patient C) lived in Port-Gentil, which has many West African immigrants. For all patients, the most likely risk factor for HIV was a heterosexual relationship with an unknown HIV-infected person. In three asymptomatic patients (A, B, and C) the HIV-2–serostatus was unexpected; in contrast, the three other patients had AIDS-related symptoms. Patients D and E had an HIV-2 Western blot pattern showing a marked decrease in anti-gag and pol reactivities compatible with their advanced stage of HIV-2 disease.

The case of a 55-year-old exclusively heterosexual asymptomatic woman (patient B) suggests the possibility of a specific variant of HIV-2 in Central Africa (2). The high frequency in primates in Gabon of natural infection with simian immunodeficiency retroviruses, which show a high degree of genetic relatedness to HIV-2 (3), could support such a hypothesis.

Two patients had typical dual reactivities to HIV-1 and HIV-2 antigens. To our knowledge, such dual reactivities have never been reported in Gabon (4). In the patient from Nigeria (patient F), the serologic pattern was typical of that usually observed in West Africa (5). Dual reactivity can result from genuine mixed infections and from serologic cross-reactivity in HIV-1 and HIV-2 infection alone; theoretically, it could also represent infection with a different, cross-reacting recombinant strain (5).

HIV-2 infection in Gabon is epidemiologically related to West Africa, because of cultural and, above all, economic ties. However, HIV-2 is not limited to immigrant populations from West Africa or to Gabonese citizens traveling in this area; it has also reached the indigenous Gabonese population. The possibility of rare cases of HIV-1 and HIV-2 coinfections, recombinant HIV-1 and HIV-2 strains, and also peculiar HIV-2 variants from Central Africa, should be considered in Gabon. A possible entry of HIV-2 infection into Central Africa from Gabon in the near future could have major public health implications.

### Acknowledgments

We thank Dr. Jean Wickings for reviewing the manuscript. CIRMF is funded by the Gabonese government, ELF Gabon, and the French Ministry of Cooperation.

**Carol Tevi-Benissan,* Madeleine Okome,† Maria Makuwa,* Moise Ndong Nkoume,† Joseph Lansoud-Soukate,* Alain Georges,* Marie-Claude Georges-Courbot,* and Laurent Belec‡**

*Centre International de Recherches Medicales, Franceville, Gabon; †Fondation Jeanne Ebori, Libreville, Gabon; and ‡Centre Hospitalo-Universitaire Broussais, Paris, France.

### References

1. World Health Organization. Global programme on AIDS. Recommendations for the selection and use of HIV antibody tests. Wkly Epidemiol Rec 1992;67:145-9.
2. Belec L, Martin PMV, Georges-Courbot MC, Brogan T, Gresenguet G, Mathiot CC, et al. Dementia as the primary manifestation of HIV-2 infection in a Central African patient. Ann Inst Pasteur Virol 1988;139:291-4.
3. Georges-Courbot MC, Moisson P, Leroy E, Pingard AM, Nerrienet E, Dubreuil G, et al. Occurrence and frequency of transmission of naturally occurring simian retroviral infections (SIV, STLV, and SRV) at the CIRMF Primate Center, Gabon. J Med Primatol 1996;25:313-26.
4. Delaporte E, Janssens W, Peeters M, Buve A, Dibanga G, Perret J-L, et al. Epidemiological and molecular characteristics of HIV infection in Gabon, 1986-1994. AIDS 1996;10:903-10.
5. Peeters M, Gershy-Damet G-M, Fransen K, Koffi K, Coulibaly M, Delaporte E, et al. Virological and polymerase chain reactions studies of HIV-1/HIV-2 dual infection in Côte d'Ivoire. Lancet 1992;340:339-40.

### Q Fever in French Guiana: New Trends

**To the Editor:** Q fever, the endemic disease caused by the rickettsial organism *Coxiella burnetii*, was first described in French Guiana in 1955 (1). Only sporadic cases were reported until 1996 when three patients were hospitalized in the intensive care unit of the Cayenne Hospital for acute respiratory distress syndrome. One of the patients died. Many cases of Q fever were diagnosed in the general population at the same time. A seroepidemiologic study was performed to determine whether the increase in cases was due to an increase in incidence or to an improvement in diagnosis. All paired samples of sera (acute-phase and convalescent-phase) from patients sent to the arbovirus laboratory for diagnosis of dengue infection from January 1, 1992, to December 31, 1996, were tested for antibodies to *C. burnetii* by immunofluorescence. All positive samples were also tested for immunoglobulin (IgM) by the same method; the IgG and IgM titers were determined by using a serial twofold dilution. A diagnosis of Q fever was made when there was a seroconversion from negative to positive or a twofold increase in IgG titer associated with the presence of IgM in the second sample.

One hundred and fifty-one of 426 paired sera collected between 1992 and 1996 were from patients recently infected with dengue fever. Twenty-five (9.1%) of 275 remaining sera were from Q fever patients. Significant differences were observed in the rates of Q fever in different years ($p < 0.01$); one (1.9%) of 53 was positive in 1992, five (9.1%) of 55 in 1993, five (8.6%) of 58 in 1994, three (4.8%) of 63 in 1995; a large increase was observed in 1996 (11 [23.9%] of 46). Differences by residence were also assessed. Rates of infection were higher in Cayenne (21 [13.0%] of 161) than in rural areas (4 [3.5%] of 114) ($p < 0.01$).

This study shows that cases of Q fever have occurred in French Guiana in recent years and that a significant increase in the incidence rate occurred in 1996. The reasons for this increase are unclear, and further studies of the epidemiology of Q fever in French Guiana are necessary. The epidemiology of Q fever is unusual in French Guiana because the rates of infection are much higher in Cayenne, the capital city, than in rural areas. No link with classical sources of infection (cattle, sheep, or goat birth products, or work in a slaughterhouse) was found. Indeed, Cayenne, with 80,000 inhabitants, is located near the Atlantic Ocean, and the prevailing winds blow from the sea. Airborne contamination from rural areas is therefore impossible. Furthermore, no large farm is in the immediate vicinity of the city. For identical reasons, contamination from the abattoirs is not likely; they are located on the west side of the city, near the Cayenne River, and the winds blow from the east. In our study, cases were almost equally distributed

